# Psychopathology in children before and after epilepsy surgery: a prospective controlled study

**DOI:** 10.1111/epi.18345

**Published:** 2025-03-12

**Authors:** Giulia Matta, Tiziana Pisano, Laura Grisotto, Flavio Giordano, Elena Cavallini, Claudia Accolla, Carmen Barba, Renzo Guerrini

**Affiliations:** ^1^ Neuroscience and Medical Genetics Department Meyer Children's Hospital IRCCS Florence Italy; ^2^ Department of Statistics, Computer Science, Application “G. Parenti” University of Florence Florence Italy; ^3^ Neurofarba Department University of Florence Florence Italy

**Keywords:** behavior, children, epilepsy surgery, outcome, psychopathology

## Abstract

**Objective:**

This study was undertaken to prospectively assess the frequency and type of psychiatric disorders (PDs) in pediatric surgical candidates and evaluate the effects of epilepsy surgery on their psychopathological profile.

**Methods:**

This is a prospective controlled study. Psychopathology was assessed using both diagnostic interviews and questionnaires completed by clinicians, parents, and whenever possible, patients, at baseline (T0) and 1 year after surgery in operated patients (T1) and 1 year after the first evaluation in a control group of nonoperated patients (T1). A “global assessment measure” was developed to integrate the results of the interviews, and the questionnaires were administered to multiple informants, at both T0 and T1. Descriptive statistics and multivariable analyses were performed for all outcomes. An ordinal logistic regression model was estimated to analyze the correlation between surgical treatment and possible changes in psychopathology at T1.

**Results:**

At T0, 103 children (42 females, mean age at first evaluation = 9.5 ± 3.92 years) with lesional epilepsy were included in the study. Thirty‐two patients (31.07%) had at least one PD, and 17 (16.5%) had more than one PD of any type. Sixty‐two (60.2%) of 103 patients were enrolled for the T1 phase of the study, of whom 39 underwent epilepsy surgery. The ordinal logistic model revealed that patients who underwent surgery and achieved Engel class I outcome were 17.57 times (*p* = .047) more likely to experience improvement in their psychopathological profile than those who were not operated on and continued to experience seizures.

**Significance:**

This prospective controlled study demonstrates a high prevalence of PD in pediatric surgical candidates and a correlation between successful surgery and reduced PD burden. An integrated approach encompassing diagnostic interviews and questionnaires, and accounting for potential disagreement between multiple informants, is essential for carefully assessing psychiatric comorbidities in children with drug‐resistant seizures.

**Key points**

- Psychopathology was assessed in 103 pediatric surgical candidates with lesional epilepsy using diagnostic interviews and questionnaires.
- At baseline, 32 patients (31.1%) had at least one PD and 17 (16.5%) had more than one PD of any type.
- Sixty‐two (60.2%) patients entered the prospective phase of the study, of whom 39 underwent epilepsy surgery and 23 served as controls.
- Seizure‐free patients after surgery were 17.57 times more likely to improve their PD than those who did not have surgery and had seizures.

## 1 INTRODUCTION

Children with drug‐resistant epilepsy are at increased risk of behavioral and emotional difficulties compared with children with other chronic diseases.^1^, ^2^ Psychiatric comorbidities may have a greater impact on health‐related quality of life than the seizures themselves, especially if unrecognized or untreated.^3^, ^4^, ^5^

Epilepsy surgery is successful in achieving seizure freedom in 64.8% of children at 1‐year follow‐up,^6^ with possible improvements in quality of life^7^, ^8^ and gains in cognitive functioning after an adequate follow‐up.^9^ Few reports have focused on the assessment of psychopathology in pediatric surgical candidates and the potential factors associated with changes in behavioral and emotional aspects after surgery.^10^, ^11^ Most studies employing questionnaires have reported improvements in behavior and emotion after epilepsy surgery,^12^, ^13^, ^14^ whereas those using the Diagnostic and Statistical Manual of Mental Disorders, (DSM) found no significant changes in the prevalence of psychiatric disorders (PD) after surgery, with some children losing and others gaining diagnoses.^15^, ^16^, ^17^ In addition, the few series^18^, ^19^, ^20^ that have included controls (usually patients with drug‐resistant epilepsy who did not undergo epilepsy surgery) have yielded contrasting results. Lendt et al.^14^ found significant improvements in internalizing, externalizing, attention, and thought problems only in the surgical group. In contrast, Tavares et al.^21^ observed improvements in scores for externalizing, thought, and attention problems in both surgical patients and controls. Finally, the only randomized controlled trial in pediatric epilepsy surgery^12^ reported a significantly greater change in Child Behavior Checklist (CBCL) total scores in the surgical than in the nonsurgical group at 1‐year follow‐up.

While assessing possible predictors of postoperative changes in the psychopathological profile, previous studies failed to find a consistent association with any preoperative or surgical variable, except for a favorable seizure outcome, which correlated with improved behavioral functioning in some surgical series.^12^, ^14^, ^18^

In addition, most studies have evaluated psychopathology only relying on parental reports, which may be biased by parents' emotional reactions to their children's illness and their expectations regarding epilepsy and psychopathology outcomes.^12^, ^13^, ^14^, ^18^, ^19^ None of the available studies has ever collected information through DSM5‐based diagnostic interviews and questionnaires from parents and children in tandem.^10^

In this prospective controlled study, we aimed to assess the prevalence, characteristics, and possible predictors of PD in 103 pediatric surgical candidates with lesional epilepsy. We then evaluated the association between epilepsy surgery and possible postoperative changes in psychopathology at 1‐year follow‐up in 39 operated children. As a control group, we included 23 patients from the same sample who did not undergo surgery during the observation period.

## 2 MATERIALS AND METHODS

The study was approved by the ethics committee of the Tuscany Region, Italy. All patients were consecutively enrolled after their first clinical assessment at Meyer Children's Hospital IRCCS, Florence, Italy. Written informed consent was obtained by parents or legal guardians.

Inclusion criteria were lesional drug‐resistant epilepsy (either focal epilepsy or developmental and epileptic encephalopathy^22^), age at baseline between 3 and 17 years, and no previous epilepsy surgery. Exclusion criteria were progressive metabolic and genetic etiology of epilepsy.

All patients underwent a presurgical workup including history, 3‐T structural magnetic resonance imaging (MRI), video‐electroencephalography (EEG), and a neuropsychological evaluation including: (1) Griffiths Mental Development Scales–Extended Revised^23^ and Wechsler Scales^24^, ^25^ for intellectual quotient (IQ)/developmental quotient (DQ) and, when indicated, Leiter International Performance Scale–Revised^26^ to measure nonverbal cognitive functioning; (2) Boston Naming Test^27^ and the word fluency test^28^ to assess language skills; and (3) digit span forward and digit span backward derived from the Wechsler scales to test verbal and working memory.

We collected information on (1) general characteristics, namely, sex, age at first assessment, and age at seizure onset; (2) seizure type and frequency at baseline (T0); (3) neuroimaging, that is, type and topography of epileptogenic lesions, if any are detectable; (4) antiseizure medications (ASMs) at baseline and 1 year after surgery in operated patients (T1) and 1 year after the first evaluation in nonoperated patients (T1); (5) seizure outcome at T1 using Engel's classification^29^ for operated patients and an equivalent of it for nonoperated patients, namely, no seizures (equivalent to Engel class I), rare seizures (equivalent to Engel class II), improved seizure frequency (equivalent to Engel class III), and unchanged or worsened seizure frequency (equivalent to Engel class IV)^9^; and (6) neuropsychological assessment, namely, IQ/DQ, language and memory performances at T0 and T1.

### 2.1 Study protocol at T0

First, we assessed psychopathology^30^ at T0 and T1 by administering to the parents/caregivers of all recruited children (1) the CBCL^31^, ^32^ and Conners' Rating Scales–Revised (CRS‐R)^33^ as standardized questionnaires and (2) the Mini International Neuropsychiatric Interview for Children and Adolescents (Minikid)^34^ for the diagnostic interviews. We also used the Behavior Rating Inventory of Executive Function–Preschool Version (BRIEF‐P)^35^ for children aged 2–5 years, because the Conners‐R is not designed for use in children younger than 3 years, and CBCL, Youth Self‐Report (YSR),^32^ and CRS‐R (adolescent version) are designed for children aged 11–17 years with IQ > 70.

In addition, we chose to use the Conners‐R exclusively to evaluate attention‐deficit/hyperactivity disorder (ADHD) symptoms and related disorders, while relying on the CBCL to assess internalizing and externalizing problems. To avoid potential confirmation bias with the Conners‐R results, we did not use the CBCL Attention Problems subscale to assess ADHD symptoms.

As a second step, we discriminated three psychopathological domains using different questionnaires, including internalizing (anxiety and depressive disorders) and externalizing problems (oppositional defiant disorder, intermittent explosive disorder and conduct disorder; CBCL and YSR) and ADHD (BRIEF‐P and CRS‐R). We considered a *T*‐score > 60 as clinically meaningful, encompassing borderline and clearly pathological scores.

We distinguished four clinical domains through the diagnostic interviews (DSM5): internalizing and externalizing problems, ADHD, and schizophrenic spectrum disorder. We used the term of “hyperactivity” to indicate a behavior characterized by hyperkinesis, impulsivity, and reduced attention span in preschool children, not identifiable as ADHD.

Finally, to ensure a comprehensive evaluation of each individual patient and to account for possible disagreements between informants, we developed a T0 “global assessment measure” that incorporated the results of the clinician, parent, and self‐report questionnaires as well as of the diagnostic interviews. This composite score at T0 was developed in a structured, multistep process. First, we determined the DSM5‐based diagnoses by integrating the clinical observation of the child and the results of the Minikid interview conducted with either the parents or the child, or both. As a second step, we scored the psychodiagnostic questionnaires, focusing on whether they exceeded the predefined cutoff values specific to each test. We then integrated the results from the diagnostic interviews and psychodiagnostic tests to derive the composite score, which categorized findings into three levels: absence of psychopathology, simple psychopathology (one PD), or complex psychopathology (more than one PD). To minimize confirmation bias, three independent evaluators, blinded to each other's evaluations, conducted the assessments. Any discrepancies among the evaluators were resolved through a consensus process.

### 2.2 Study protocol at T1

To assess possible changes in the psychopathological profile at T1, we calculated the reliable change index (RCI)^36^, ^37^ for each domain that emerged from the questionnaires. We established a confidence interval of 90% to define clinically significant changes.

To ensure consistency in scoring and account for possible discrepancies between informants and between diagnostic interviews and questionnaires, we categorized the psychopathological profile at T1 as improved, worsened, or unchanged based on a T1 global assessment measure that integrated the RCI for each domain and the DSM5‐based diagnoses at T1.

As an external validation, all the results were independently validated by two reviewers blinded to the clinician's assessments.

### 2.3 Statistical analysis

We performed a descriptive statistical analysis to summarize the variables of interest as follows: family history of psychopathology, seizure type and frequency in the 6 months preceding the evaluation, age at first assessment, age at seizure onset, type and topography of epileptogenic lesion, if any, and ASMs at T0 and T1. Specifically, categorical variables were summarized as number (percentage), whereas continuous variables were presented as mean ± SD.

We analyzed the relationship between the variables of interest and the evidence for, and severity of, psychopathology at T0 and T1 using Pearson *χ* ^2^ test of independence or Fisher exact tests on tables of frequency for each categorical variable of interest and Student *t*‐test or analysis of variance for each continuous variable of interest, where appropriate.

Additionally, we calculated Cohen kappa^38^ to assess the degree of agreement between different informants (parents, patients, and clinician) regarding the diagnosis of internalizing disorders, externalizing disorders, and ADHD, in the hyperactivity–impulsivity, inattention, and combined forms as defined by the questionnaires.

Finally, to evaluate the correlation between surgical treatment and possible changes in psychopathology at T1, we estimated an ordinal logistic regression model with age at epilepsy onset, change in ASM at T1, and severity of psychopathology at T0 as confounders. We tested the seizure outcome (Engel class IA vs. Engel class IB–IV) as a possible effect modifier and defined appropriate models based on the results of the analysis.

We carried out all statistical analyses using Stata Statistical Software version 18.0 (StataCorp, 2018).

## 3 RESULTS AT BASELINE

### 3.1 Population at T0

According to the inclusion and exclusion criteria, we recruited 103 pediatric surgical candidates (42 females, mean age at evaluation = 9.5 ± 3.92 years, range = 3–16.2 years) at the Neuroscience and Medical Genetics Department, Meyer Children's Hospital IRCCS, Florence, Italy. We found a positive family history of PDs in 10 of 96 patients (9.71%); the remaining seven children were adopted (Table 1).

**TABLE 1:** Descriptive analysis of the study sample at T0.

Characteristic	*n*	%	*p* Surgical vs. control group
Sex			
F	42	40.8	.694
M	61	59.2	
Age at first evaluation, years	Mean = 9.5	SD = 3.92	.213
Age at seizure onset, years	Mean = 4.9	SD = 4.01	.427
Family history of psychiatric disorders			
No	86	83.5	.717
Yes	10	9.7	
Missing value	6	5.8	
Seizure frequency			
Daily	55	53.4	.369
Weekly	25	24.3	
Monthly	19	18.4	
No seizures	4	3.9	
Type of epilepsy			
Focal	59	57.3	.441
Focal to bilateral	22	21.4	
DEE	22	21.4	
ASM			
Polytherapy	79	76.7	.642
One ASM	24	23.3	
Side of lesion			
R	43	41.7	.596
L	55	53.4	
Bilateral	3	2.9	
Midline	2	1.9	
Topography of lesion			
Unilobar temporal	39	37.9	.243
Unilobar frontal	34	33.0	
Unilobar parietal	7	6.8	
Unilobar occipital	2	1.9	
Multilobar	19	18.4	
Hypothalamic	2	1.9	
Neuropsychological assessment			
Normal IQ/DQ	52	50.5	.973
Mild ID	22	21.4	
Moderate ID	19	18.4	
Severe ID	10	9.7	
IQ/DQ	Mean = 79.58	SD = 23.43	.275
Memory			
Normal	45	60.0	.929
Mild deficit	14	18.7	
Moderate deficit	11	14.7	
Severe deficit	5	6.7	
Total	75	100.0	
NA	28	27.2	
Language			
Normal	56	57.1	.491
Mild deficit	21	21.4	
Moderate deficit	9	9.2	
Severe deficit	12	12.2	
Total	98	100.0	
NA	5	4.9	
Psychopathology by DSM5 criteria			
No	54	52.4	.062
ADHD	18	17.5	
Internalizing disorders	15	14.6	
Externalizing disorders	15	14.6	
Schizophrenic spectrum disorder	1	1.0	

Abbreviations: ADHD, attention‐deficit/hyperactivity disorder; ASM, antiseizure medication; DEE, developmental and epileptic encephalopathy; DQ, developmental quotient; DSM5, Diagnostic and Statistical Manual of Mental Disorders, fifth edition; F, female; ID, intellectual disability; IQ, intellectual quotient; L, left; M, male; NA, not available; R, right.

Fifty‐five patients (53.4%) had daily seizures, 25 (24.27%) had weekly seizures, and 19 (18.45%) had monthly seizures. The remaining four patients (3.88%), after previous drug resistance, did not experience any seizure in the 6 months preceding the evaluation. Fifty‐nine patients (57.28%) exhibited focal seizures, 22 (21.36%) focal to bilateral seizures, and 22 (21.36%) developmental and epileptic encephalopathies.^22^ Nineteen patients with developmental and epileptic encephalopathies had epileptic spasms syndrome,^39^ with or without focal seizures, and the remaining three also experienced tonic seizures in addition to epileptic spasms.^40^ All showed clear focal clinical and EEG features, correlated with MRI‐visible structural brain abnormality. Seventy‐nine patients were on polytherapy (76.7%), whereas the remaining 24 (23.30%) were on a single ASM. All patients were classified as lesional; the structural abnormality on brain MRI was on the left hemisphere in 55 patients (53.4%), on the right in 43 patients (41.75%), bilateral in the context of tuberous sclerosis complex in three (2.91%), and in the midline in two (1.94%). The epileptogenic lesion was unilobar temporal in 39 (37.86%) patients, unilobar frontal in 34 (33.01%), unilobar parietal in seven (6.8%), unilobar occipital in two (1.94%), multilobar in 19 (18.4%) with involvement of the temporal lobe in 13 (12.62%), and hypothalamic in two (1.94%). Neuroimaging revealed suspected malformations of cortical development (MCD) in 65 patients (63.11%), gliotic scars in 18 (17.48%), suspected tumors in 13 (12.62%), cortical tubers in three (2.91%), and hippocampal sclerosis and hypothalamic hamartoma in two patients each (1.94%).

Fifty‐two patients (50.48%) had a normal cognitive level, 22 (21.35%) mild intellectual disability (ID), 19 (18.44%) moderate ID, and 10 (9.70%) severe ID. The full‐scale IQ varied between 40 and 136, with an average index of 79.58.

Of the 75 children (72.81%) in whom the evaluation of the memory function was possible, 45 (60%) had normal scores, 14 (18.67%) a mild deficit, 11 (14, 67%) a moderate deficit, and five (6.67%) a severe deficit. The working memory index ranged between 52 and 115, with an average of 77.04.

Of the 98 patients (95.14%) in whom it was possible to examine language functions, 56 (57.14%) had normal scores; 21 (21.43%) exhibited a mild deficit, nine (18%) a moderate deficit, and 12 (12.24%) a severe deficit. The verbal intellectual component or verbal intellectual quotient varied between 46 and 134, with an average index of 82.16.

### 3.2 Psychopathology at T0

The results of diagnostic interviews and structured questionnaires in both parents and children are detailed in Results S1 and Table 1.

#### 3.2.1 Prevalence and severity of psychopathology at T0

Based on the T0 global assessment measure, 32 patients (31.07%) had at least one PD and 17 (16.5%) had more than one PD of any type.

#### 3.2.2 Analysis of associations between clinical variables and psychopathology

The analysis of association failed to reveal a significant correlation between any of the epilepsy and clinical variables of interest and psychopathology or its severity.

Conversely, cognitive level and language performances expressed as categorical variables (normal level, mild, moderate, and severe impairment) correlated significantly with psychopathology severity (*p* = .045 and *p* < .0001, respectively).

## 4 RESULTS AT T1

### 4.1 Population at T1

Sixty‐two patients were included in the analysis at T1, of whom 39 underwent surgical treatment. Surgical and control patients did not differ significantly with respect to clinical, neuropsychological, and neuroimaging features (see Table 1). The 23 controls did not undergo surgery by 1‐year follow‐up for the following reasons: seizure improvement during the presurgical evaluation, need for further presurgical investigations (e.g., stereoelectroencephalography) that delayed surgery or ruled it out, and in most patients, parents' decision to wait for surgery for fear of possible functional or cognitive worsening. ASMs were modified in 28 of 62 patients (41.16%).

#### 4.1.1 Control group at T1

Of the 23 control patients, 18 (78.26%) harbored MRI changes consistent with MCD, four (17.39%) with gliotic scars and one (4.34%) with hippocampal sclerosis.

Concerning seizure outcome, we observed Engel I equivalent in four patients (17.39%) and Engel IV equivalent in 19 patients (82.61%).ASM were modified , either in dosage or type of ASM, in 15 patients of the control group. Specifically, three of the four seizure‐free patients, medical treatment was not modified, whereas in the remaining one the dosage of ASM was increased.

#### 4.1.2 Surgical group at T1

Of the 39 operated patients, 18 (46.15%) underwent lesionectomy, 16 (41.03%) unilobar lobectomy, and five (12.82%) multilobar lobectomy.

The anatomic site of surgery was temporal in 18 patients (46.1%), frontal in 16 (41.03%), temporo‐occipital in three (7.7%), and parietal and hypothalamic in one (2.56%) each.

Histopathology revealed (1) MCD in 22 patients (56.4%), including FCD I in 15 (38.4%), FCD IIa and FCD IIb in three (7.7%) each, and FCD IIIa in one (2.56%); (2) tumors in 11 patients (28.2%), including ganglioglioma World Health Organization (WHO) I and dysembryoplastic neuroepithelial tumor in three patients (7.7%) each, polymorphous low‐grade neuroepithelial tumor, astrocytoma WHO I, ependymoma WHO II, pleiomorphic xanthoastrocytoma, and multinodular and vacuolating neuronal tumor in one (2.56%) each; (3) gliotic scars in three (7.7%); (4) cortical tuber in two patients (5.13%); and (5) a dermoid cyst in one patient (2.56%).

Seizure outcomes included Engel class I in 30 patients (76.92%), Engel II in three patients (7.69%), and Engel IV in six patients (15.38%). ASMs were increased in three patients and reduced in 10 in the surgical group.

### 4.2 Psychopathological outcome at T1

The results of diagnostic interviews and of the RCI at T1 are detailed in Results S1.

#### 4.2.1 Prevalence and severity of psychopathology at T1

Based on the global assessment measure, 20 patients (32.26%) had at least one PD and nine (14.52%) had more than one PD of any type.

#### 4.2.2 Analysis of associations between clinical variables and psychopathology

Cognitive level and memory and language performance expressed as categorical variables correlated significantly with the severity of psychopathology (*p* < .0001, *p* = .005, and *p* < .0001, respectively).

#### 4.2.3 Analysis of correlation between clinically meaningful changes in psychopathology and epilepsy surgery

**FIGURE 1:**
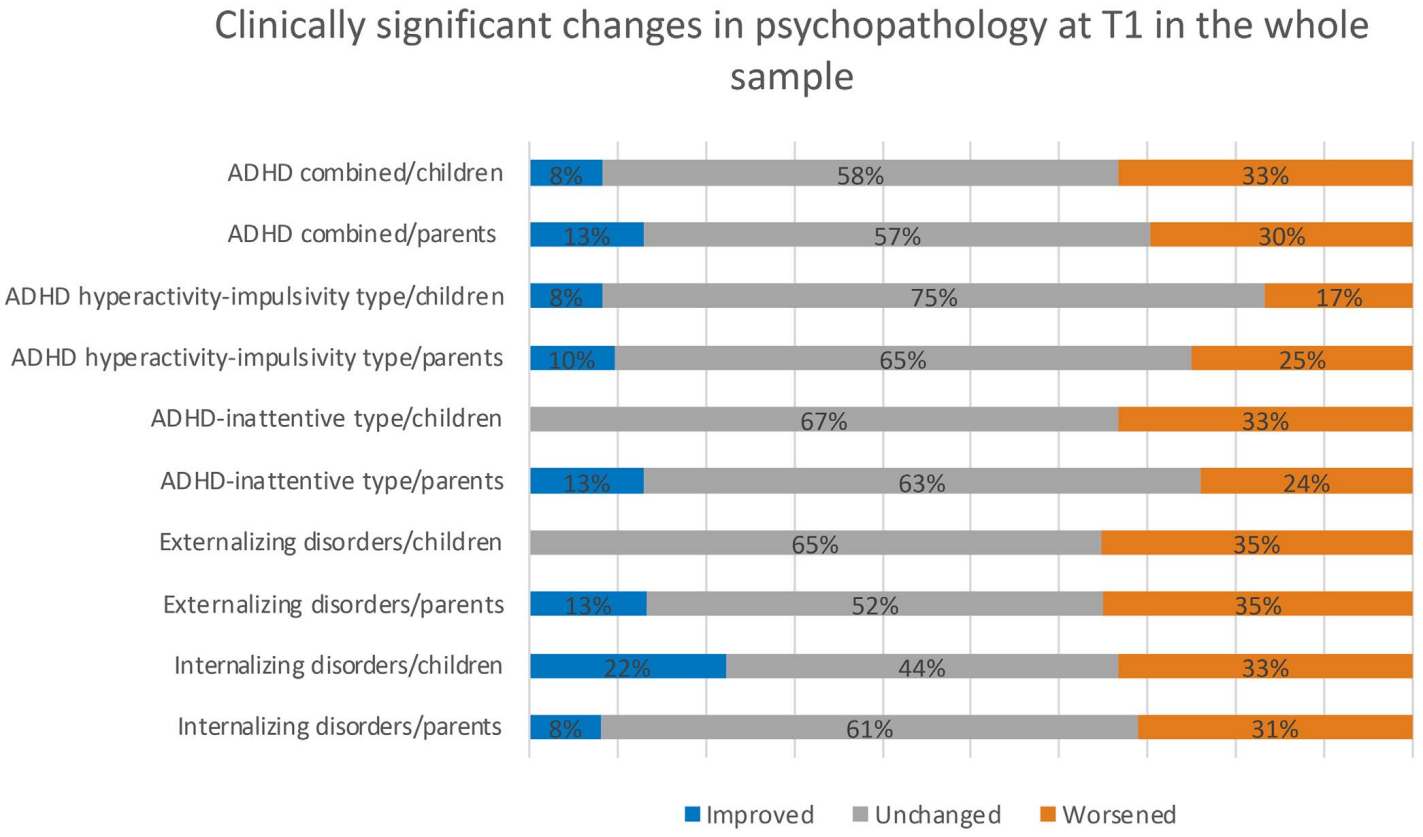
Distribution of the changes in the psychopathological profile as measured by reliable change index for the whole sample. ADHD, attention‐deficit/hyperactivity disorder.

**FIGURE 2:**
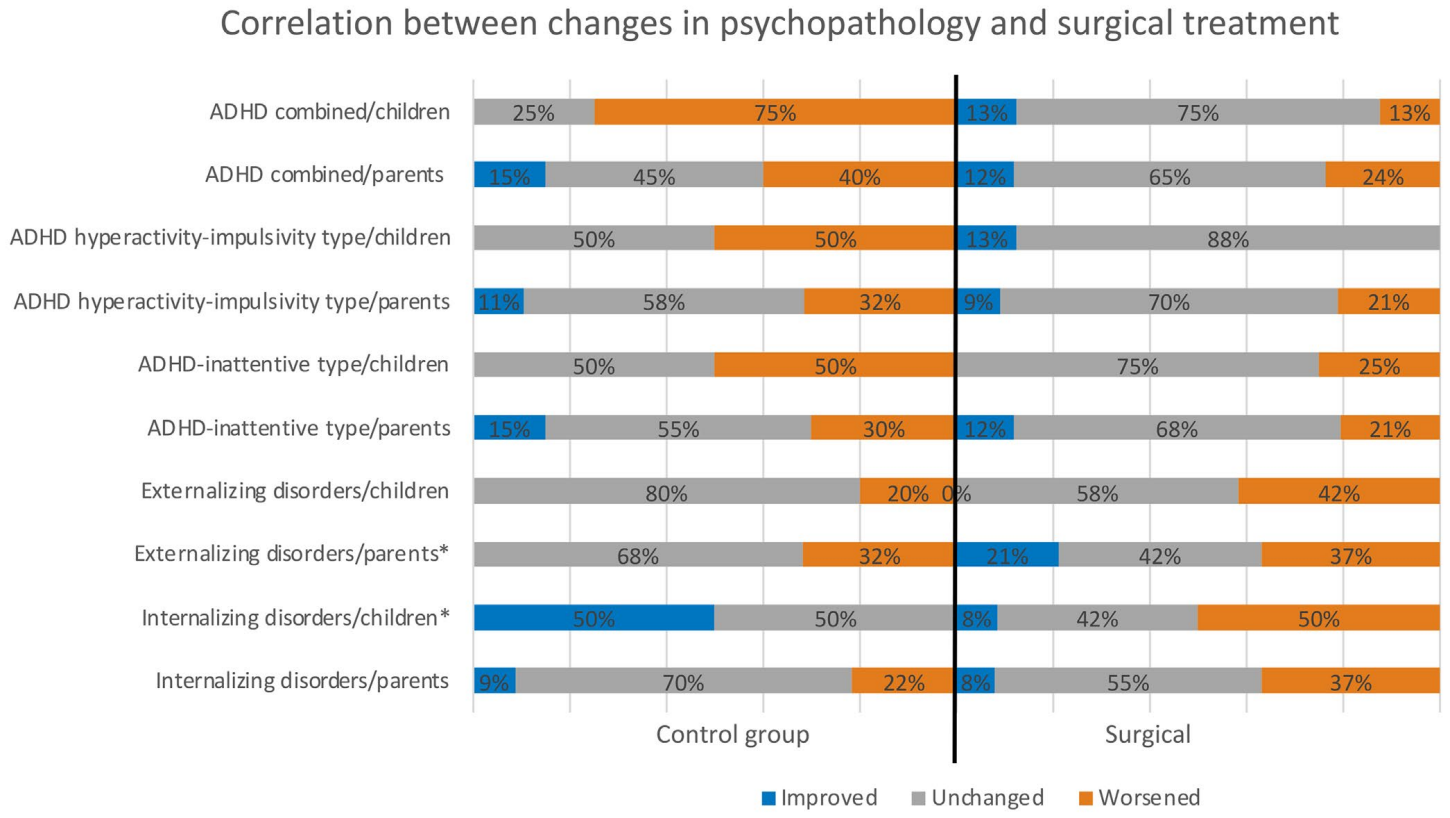
Distribution of the changes in the psychopathological profile for the surgical and control groups. ADHD, attention‐deficit/hyperactivity disorder. Note: Significant results are indicated with an asterisk.

The distribution of changes in the psychopathological profile as measured by the RCI (categorical variables) is shown in Figure 1 for the whole sample and in Figure 2 for the surgical and control groups.

Epilepsy surgery correlated significantly (*p* = .045) with clinically meaningful changes in self‐reported internalizing problems, but not with clinically meaningful changes in parent‐reported internalizing problems (*p* = .462).

Additionally, we observed a significant correlation (*p* = .038) between the variable epilepsy surgery and clinically meaningful changes in parent‐reported externalizing problems. However, there was no correlation between clinically meaningful changes in self‐reported externalizing problems and surgical treatment (*p* = .394).

We found no significant correlations between the variable intervention and clinically meaningful changes in the three types of ADHD as reported by both parents and patients themselves.

### 4.3 Ordinal logistic regression model

By estimating the crude effect of surgery, adjusted only for possible confounders, we found that patients who underwent surgery were 4.49 (*p* = .016) times more likely to have an improvement in their psychopathological profile than those not operated on (Table 2).

**TABLE 2:** Ordinal logistic regression models.

Model		OR	95% CI		*p*
Model for crude effect of surgery					
Surgery		4.495	1.330	15.184	.**016**
Psychopathology T0	No disorder				
1	Simple	5.065	1.350	19.007	.**016**
2	Complex	8.249	1.750	38.881	.**008**
Modification of ASMs at T1	No				
	Yes	1.701	.527	5.490	.374
Age at seizure onset		.993	.876	1.125	.907
Model for Engel I patients					
Surgery		17.566	1.040	296.602	.**047**
Psychopathology T0	No disorder				
1	Simple (1 type of disorder)	18.456	2.162	157.570	.**008**
2	Complex (>1 type of disorder)	11.883	1.209	116.775	.**034**
Modification of ASMs at T1	No				
	Yes	4.367	.629	30.304	.136
Age at seizure onset		1.042	.884	1.228	.624
Model for Engel II–IV patients					
Surgery		.932	.148	5.866	.940
Psychopathology T0	No disorder				
1	Simple (1 type of disorder)	1.405	.204	9.700	.730
2	Complex (>1 type of disorder)	7.439	.699	79.154	.096
Modification of ASMs at T1	No				
	Yes	.523	.083	3.293	.490
Age at seizure onset		.751	.560	1.006	.055

*Note*: Significant values are in bold.

Abbreviations: ASM, antiseizure medication; CI, confidence intervals; OR, odds ratio.

We observed a significant interaction between surgical treatment and seizure outcome, with patients who underwent surgery resulting more frequently in Engel class I (odds ratio [OR] = 6.55, *p* = .008) than patients who were not operated on.

As a subsequent analysis, we estimated two separate models, one for Engel I patients and the other for Engel II–IV patients. In the first model, patients who underwent surgery were 17.57 (*p* = .047) times more likely to have an improvement in their psychopathological profile than patients who did not undergo surgery, whereas in the second model, there was no significant association between surgery and psychopathological outcome at T1 (OR = .93, *p* = .940).

Regarding the effect of confounders, only the severity of psychopathology at T0 correlated positively with an improved psychopathological profile at T1.

## 5 DISCUSSION

In this prospective controlled study, we used a multi‐informant approach to assess prevalence and severity of PD in a cohort of pediatric surgical candidates. We then assessed whether a correlation exists between epilepsy surgery and postoperative changes in psychopathology and used nonoperated patients from the initial cohort as a control group.

At T0, integrating the results of self‐report and parent questionnaires and DSM5‐based diagnoses, we found that 32 patients (31.07%) had at least one PD and 17 (16.5%) had more than one PD of any type. Previous studies^14^, ^15^, ^16^, ^17^, ^41^ reported the prevalence of psychopathology in surgical candidates to vary between 39%^14^ and 80%.^10,41^ However, these figures cannot be compared with those emerging from our study due to differences in inclusion criteria, type of psychodiagnostics assessment, and time of observation.

In this study, the interpretation of questionnaires is further challenged by significant discrepancies in the ratings provided by the different informants (i.e., clinicians, parents, and patients).

Specifically, clinician–parent agreement was fair for the diagnoses of internalizing and externalizing disorders and for ADHD in the hyperactivity–impulsive form and in the combined form but limited for the diagnosis of ADHD in the inattentive form. The observation that parents were more likely than the clinician to report attention problems is possibly related to the additional information they received from school and recreational activities.

There was substantial clinician–patient agreement in the diagnosis of ADHD in the hyperactivity–impulsivity form and moderate agreement in the diagnosis of internalizing disorders. Patients could easily identify their own problems of hyperactivity and impulsivity because of the negative impact on daily activities and sufficient awareness about their own emotional distress. However, they were unable to recognize their own externalizing problems, which often have more detrimental impact on clinicians and parents than on the patients themselves.

We found substantial parent–patient agreement on internalizing problems, although in cases of disagreement it was the parent who identified a disorder not recognized by the patient. Parents were more likely to identify emotional disturbances in their children, even when compared to the clinician. Accordingly, Berg et al.^42^ highlight how parents' concerns, expectations, and personal psychiatric issues can lead to an overestimation of their children's internalizing disorders.

Taken together, these observations highlight the need to gather information from multiple informants, and especially from the patients themselves, whenever possible, to comprehensively assess their psychopathological profile.

As a second step, we analyzed the predictors of psychopathology at T0 and found that linguistic deficits and cognitive impairment were the only factors associated with the severity of psychopathology in surgical candidates. This observation is in line with previous studies^19^, ^43^ that emphasized the role of communicative functions in ensuring the correct acquisition of a sense of self in relation to peers, and the ability to express one's emotions.^44^ In addition, drug‐resistant seizures and their treatment may negatively affect language development and indirectly contribute to precipitate new PD or worsen pre‐existing conditions. For this reason, it is important to carefully assess language functions in children with drug‐resistant seizures to provide timely rehabilitative interventions. Among the clinical variables, the etiology and topography of the lesions were quite heterogeneous, in line with previous pediatric surgical studies.^6^ This variability might have influenced the actual magnitude of their correlation with PD changes.

While assessing the predictors of psychopathology at T1, we observed a significant correlation between clinically significant changes in both the internalizing and externalizing problems and epilepsy surgery, with a striking difference in the proportion of operated patients in whom PD improved, compared to control nonsurgical patients. Previous studies yielded conflicting results on this issue. Some authors highlighted the correlation between postoperative changes in psychopathology and the intervention itself,^12^, ^14^, ^18^ others emphasized the relationship with seizure outcome,^20^ and others found no measurable effect of surgery and seizure freedom on PD.^15^, ^16^, ^17^, ^45^

As an additional source of complexity, parents were more likely to report postoperative improvements in internalizing and externalizing problems than patients, as also reported in other studies.^21^ Another crucial methodological aspect is that the RCI applies exclusively to the questionnaires, thus excluding any additional insights provided by diagnostic interviews. Tavares et al.,^21^ who evaluated the long‐term evolution of anxiety and depressive symptoms in surgical patients and in a control nonsurgical group, found similar self‐reported depressive and anxiety manifestations in both groups. However, parental reports indicated that children with ongoing seizures exhibited significantly more anxiety/depressive symptoms compared to those who were seizure‐free.

To overcome these limitations, we employed a global assessment measure that integrated both the RCI and the diagnostic interviews in the ordinal logistic regression model, while accounting for possible disagreement among multiple informants. Our analysis revealed that patients who underwent surgery and achieved Engel class I outcome were 17.57 times more likely to experience an improvement in their psychopathological profile compared to patients who did not undergo surgery and failed to achieve seizure freedom with medical therapy alone, regardless of the other variables of interest.

Our study has several limitations. First, the neuropsychological and psychodiagnostics assessments were not double‐blind, with the clinician's judgment potentially influenced by their being aware of the therapeutic process. Our results might be affected by the small sample size and short postoperative follow‐up.^10^ In addition, because most patients were still on ASM 1 year after surgery, the potential beneficial effect of discontinuing ASM on cognitive and behavioral outcomes in the operated children^9^, ^20^, ^45^, ^46^, ^47^ remains undefined. Finally, there was a striking difference in seizure outcome between operated and nonoperated patients, as 76.92% of those in the surgical group were in Engel class I,^7^, ^8^ whereas only 17.4% of the patients in the control group were seizure‐free at T1. Given that our primary aim was to evaluate the overall effectiveness of the intervention rather than to investigate specific predictors of postoperative outcomes, we opted not to develop additional models focused on seizure freedom in the subgroup of patients undergoing surgery. Exploring the relationship between seizure freedom and PD reduction might have enhanced the understanding of the contributing factors to global postoperative improvements.

Despite these limitations, our study demonstrates a high prevalence of PD in pediatric surgical candidates and a correlation between successful surgery and improvement in psychopathology. Whereas some studies have reported possible behavioral worsening in adults^48^, ^49^ and general stability in most children^10^ after epilepsy surgery, our findings demonstrate that improvement in PD, albeit with a degree of unpredictability, is significantly more likely than deterioration. Therefore, a PD should not be considered as a contraindication to epilepsy surgery.

Our results underline the need to promptly refer children with drug‐resistant epilepsy for a comprehensive preoperative neuropsychological assessment, including psychodiagnostics.^2^ This approach might help identify patients with higher chances to benefit from early surgical intervention and optimize the management of psychiatric comorbidities before and after surgery.

## AUTHOR CONTRIBUTIONS

**Giulia Matta:** Data acquisition; analysis and interpretation; drafting of the paper. **Tiziana Pisano:** Design of the work; data analysis and interpretation; revision of the paper. **Laura Grisotto:** Statistical analysis; drafting of the paper. **Flavio Giordano:** Surgical data interpretation; revision of the paper. **Elena Cavallini:** Data acquisition and analysis; revision of the paper. **Claudia Accolla:** Neuropsychological data analysis and interpretation; revision of the paper. **Carmen Barba:** Conception and design of the work; data analysis and interpretation; drafting of the paper. **Renzo Guerrini:** Design of the work; data interpretation; revision of the paper.

## FUNDING INFORMATION

This research has been funded by the European Union‐Next Generation EU, Mission 4 Component 1, CUP 53D23018390006 (Bando PRIN, project code 2022FMTET to C.B.).

## CONFLICT OF INTEREST STATEMENT

None of the authors has any conflict of interest to disclose. We confirm that we have read the Journal's position on issues involved in ethical publication and affirm that this report is consistent with those guidelines.

## Supporting information


Data S1.

## Data Availability

All data relevant to the study are included in the article or tables. Additional data are available from the corresponding author upon reasonable request.
