# Supplementary material for: Psychopathology in children before and after epilepsy surgery: a prospective controlled study
Source: Epilepsia. 2025 Mar 12;66(6):1876–87. doi: 10.1111/epi.18345 (PMC12169412; doi:10.1111/epi.18345)
Supplement: Supplementary file 1 — Data S1. [file EPI-66-1876-s001.docx]

***Supplemental Results***

***Psychopathology at T0***

*Results of the diagnostic interviews*

PD were found in 49 patients (47.57%) with the following distribution: ADHD in 18 patients (36.7%, of whom six under five years age exhibited “hyperactivity”, a sign of atypical neurodevelopment which suggest further evolution in ADHD at scholar age), internalizing problems in 15 (30.6%, depressive disorder in 13 and anxiety disorder in two), externalizing disorders in 15 (oppositional-defiant disorder (DOP) in 14 and conduct disorder in one) and schizophrenic spectrum disorder in one (0.97%).

*Results of the questionnaires administered to different informants*

The CBCL administered to parents revealed: a) internalizing problems in 44 patients (42.72%), with a T score ranging from 29 to 85 (mean 56.65, SD 10.29) and b) externalizing problems in 19 (18.45%), with a T score ranging from 33 to 71 (mean 52.13, SD 9.0).

CPR-S administered to parents revealed: a) ADHD, inattention form, in 46 patients (48.42%), with a T score ranging between 37 and 99 ( mean of 59.63, SD 14.42); b) ADHD, hyperactivity-impulsivity form in 17 (17.89%), with a T score ranging between 36 and 88, (mean 51.68, SD 12.36) and c) combined ADHD in 30 (31.58%), with a T score ranging between 36 and 92 (mean 56.27, SD 14.02),

CPR-S administered to the patients themselves indicated: a) ADHD, inattention form, in three patients (13.04%), with a T score ranging between 35 and 72 ( mean 50.04,SD 11.12); b) ADHD, hyperactivity-impulsivity form in one (4.55%), with a T score ranging between 35 and 61 (mean 43.72 (SD 7.94) and c) combined ADHD in three (13.64%), with a T score ranging between 35 and 69 ( mean 46.5, SD 10).

The YSRs administered to 29 patients revealed: a) internalizing problems in eight patients (27.59%), with an average T score ranging between 32 and 80 (mean 52.24, SD 11.45) and b) externalizing problems in only one child (3.45%), with a T score ranging between 29 and 67 (mean T score 46.72, SD 8.77).

The Brief-P test administered to 19 patients aged less than six years detected executive dysfunction (clinical scores in the Working Memory, Inhibition and ISCI indices) in seven of them (36.84%).

*Analysis of level of concordance between different informants*

When analyzing the level of concordance between parents and examiner, we found a fair concordance value in the diagnosis of internalizing (Cohen's kappa = 0.2855) and externalizing disorders (Cohen's kappa = 0.2974, ADHD hyperactivity-impulsivity form (Cohen's kappa = 0.2836) and ADHD combined form (Cohen's kappa = 0.2554). Conversely, the concordance value for ADHD inattentive form was slight (Cohen's kappa = 0.1190).

When analyzing the level of concordance between patients and the examiner, we found a moderate agreement value in the diagnosis of internalizing disorders (Cohen's kappa = 0.4183), a fair agreement value in the diagnosis of ADHD inattentive (Cohen's kappa 0.3301) and combined form (Cohen's kappa = 0.3265), and a substantial concordance value in the diagnosis of ADHD hyperactivity-impulsivity form (Cohen's kappa = 0.6452). There was no agreement in the diagnosis of externalizing disorders.

When analyzing the level of concordance between patients and parents, we found a substantial agreement value in the diagnosis of internalizing disorders (Cohen's kappa = 0.6384), a moderate agreement in the diagnosis of ADHD hyperactivity-impulsivity form (Cohen's kappa = 0.4634) and a fair agreement for that of ADHD combined form (kappa of Cohen = 0.3973). Agreement was slight for the diagnosis of ADHD inattentive form (Cohen's kappa 0.1712) and absent for the diagnosis of externalizing disorders.

*Treatment of PD at T0*

Of the nine patients with a previous diagnosis of ADHD/hyperactivity, none received targeted drug therapy, and only one had undergone psychoeducational intervention. ADHD was diagnosed in nine additional patients during presurgical evaluation.

All 15 patients with internalizing problems (two with anxiety disorder and 13 with depressive mood disorder) had a definite diagnosis. None were receiving targeted drug therapy, but 10 (two with anxiety disorder and eight with depressive mood disorder) had received psychotherapy.

Similarly, all 15 patients with externalizing problems (one with conduct disorder and 14 with oppositional-defiant disorder) had a known diagnosis. None were receiving targeted drug therapy, and five (all with oppositional-defiant disorder) had undergone psychoeducational intervention.

The patient with schizophrenic disorder was diagnosed during presurgical evaluation, but targeted drug therapy was not initiated due to parental refusal.

***Psychopathology at T1***

*Results of the diagnostic interviews*

PD were diagnosed in 16 out of 39 patients of the surgical group (41.02%) as follows: ADHD in seven patients (17.94%, of which four under five years age exhibited “hyperactivity”), externalizing disorders in five (12.82%, oppositional-defiant disorder in all), internalizing problems in three (7.69%, depressive disorder in all), and schizophrenic spectrum disorder in one (4,35%).

PD were found in 13 out of 23 patients of the control group (56.52%) as follows: ADHD in six patients (26.09%, of which one under five years age exhibited “hyperactivity”), externalizing disorders in four (17.39%, oppositional-defiant disorder in all) and internalizing problems in three (13.04%, depressive disorder in two and anxiety disorder in one).

*Results of the questionnaires administered to different informants*

Surgical group

The CBCL administered to parents revealed: a) internalizing problems in 10 patients (26.31%), with a T score ranging from 33 to 74 (mean 52.42, SD 10.87) and b) externalizing problems in nine patients (23.68%), with a T score ranging from 34 to 71 (mean 51.31, SD 10.95)

CPR-S administered to parents indicated: a) ADHD, inattentive form, in 13 patients (36.11%), with a T score ranging between 38 and 89 ( mean of 56.05, SD 15.25); b) ADHD, hyperactivity-impulsivity form in nine patients (25%), with a T score ranging between 36 and 93, (mean 50.80, SD 14.06) and c) ADHD combined form, in eight patients (22.2%), with a T score ranging between 37 and 94 (mean 53.02, SD 14.59),

CPR-S administered to patients themselves indicated: a) ADHD, inattentive form, in one patient (8,33%), with a T score ranging between 35 and 68 (mean 44.42, SD 9.8), and b) ADHD combined form in one patient (8.33%), with a T score ranging between 35 and 64 (mean 41.67, SD 7.9). ADHD, hyperactivity-impulsivity form was not observed in any patient.

The YSRs administered to patients: a) internalizing problems in four patients (33,33%), with an average T score ranging between 30 and 67 (mean 51.58, SD 12.31) and b) externalizing problems in two children (16.67%), with a T score ranging between 29 and 60 (mean T score 45, SD 9.42).

The Brief-P test administered to eight patients of the surgical group aged less than six years detected executive dysfunction (clinical scores in the Working Memory, Inhibition and ISCI indices) in six of them (75%).

Control group

The CBCL administered to parents revealed: a) internalizing problems in seven patients (30.43%), with a T score ranging from 41 to 73 (mean 53.13, SD 8.81) and b) externalizing problems in three patients (13.64%), with a T score ranging from 34 to 68 (mean 49.27, SD 9.49).

CPR-S administered to parents indicated: a) ADHD, inattentive form, in 11 patients (52.38%), with a T score ranging between 40 and 99 ( mean of 61.67, SD 15.96); b) ADHD, hyperactivity-impulsivity form in two patients (9.52%), with a T score ranging between 39 and 65, (mean 48.71, SD 6.94) and c) ADHD combined form, in three patients (14.28%), with a T score ranging between 22 and 83 (mean 53.76, SD 12.88),

CPR-S administered to patients themselves did not reveal ADHD in any patient.

The YSRs administered to patients indicated internalizing problems in four patients (54.17%), with an average T score ranging between 36 and 75 (mean 57.14, SD 12.73).

The Brief-P test administered to two patients aged less than six years detected executive dysfunction (clinical scores in the Working Memory, Inhibition and ISCI indices) in one of them (50%).

*Results of RCI on the questionnaires*

By calculating the RCI based on the scores from the questionnaires administered to the parents, we could observe:

1. In relation to the *internalizing problems* of the 61 evaluable patients:
   1. clinically meaningful improvement in five patients (two control and three surgical group),
   2. non-clinically significant changes in 37 (16 control and 21 surgical group)
   3. worsening in 19 patients (five control and 14 surgical group);
2. In relation to the *externalizing problems* of the 60 assessable patients:
   1. a clinically meaningful improvement in eight patients (eight surgical group),
   2. non-clinically significant changes in 31 (15 control and 16 surgical group),
   3. a worsening in 21 patients (seven control and 14 surgical group);
3. in relation to *ADHD, it forms inattention* of the 54 evaluable patients:
   1. a clinically meaningful improvement in seven patients (three control and four surgical group),
   2. non-clinically significant changes in 34 (11 control and 23 surgical group),
   3. a worsening in 13 patients (six control and seven surgical group);
4. in relation to *ADHD, the hyperactivity form impulsivity* of the 54 evaluable patients:
   1. a clinically meaningful improvement in five patients (two control and three surgical group),
   2. non-clinically significant changes in 36 (11 control and 25 surgical group),
   3. a worsening in 13 patients (seven control and six surgical group);
5. in relation to *ADHD combined form* of the 54 evaluable patients:
   1. a clinically meaningful improvement in seven patients (three control and four surgical group),
   2. non-clinically significant changes in 31 (nine control and 22 surgical group),
   3. a worsening in 16 patients (eight control and eight surgical group).

By calculating the RCI based on the scores from the questionnaires administered to the *patients*, we could observe:

1. As regards *internalizing problems*, in the 18 evaluable patients:
   1. clinically meaningful improvement in four patients (three control and one surgical group),
   2. non-clinically meaningful changes in eight (three control and five surgical group),
   3. worsening in 6 patients (six surgical group);
2. As regards *externalizing problems*, in the 17 evaluable patients:
   1. non-clinically meaningful changes in 11 (four control and seven surgical group),
   2. worsening in six patients (one control and five surgical group);
3. as far as *ADHD is concerned, it forms inattention*, in the 12 evaluable patients:
   1. non-clinically meaningful changes in eight (two control and six surgical group),
   2. worsening in four patients (two control and two surgical group);
4. as regards *ADHD, the form of hyperactivity impulsivity*, in the 12 evaluable patients:
   1. a clinically meaningful improvement in one patient (one surgical group),
   2. non-clinically significant changes in nine (two control and seven surgical group),
   3. a worsening in two patients (two control group);
5. as regards *the combined form of ADHD*, in the 12 evaluable patients:
   1. a clinically meaningful improvement in one patient (one surgical group),
   2. non-clinically significant changes in seven (one control and six surgical group),
   3. a worsening in four patients (three control and one surgical group).

*Psychopathological outcome in the surgical group*

We compared psychopathological outcomes (improved, unchanged, or worsened) between patients between patients who achieved class Engel IA and those who were in class IB-IV. We did not find any significant difference between these two subgroups in terms of outcome measures.

In addition, among the non-seizure free patients, four showed improvement in PD symptoms at T1.

*Treatment of PD at T1*

Only the one patient diagnosed with the schizophrenic disorder, who had not been previously treated, began risperidone treatment six months after surgery. This treatment did not result in any psychopathological improvement at T1. The remaining patients had no changes in PD treatment between T0 to T1.
